# Dignity as recognition respect: lessons from the cases of Charlie Gard, Alfie Evans and Isaiah Haastrup

**DOI:** 10.1186/s12910-026-01506-3

**Published:** 2026-06-16

**Authors:** Nanette Ryan

**Affiliations:** Centre for Biomedical Ethics, National University of Singapore, Singapore, Singapore

**Keywords:** Dignity, Recognition respect, Best interests, Withdrawal of treatment, Paediatrics, Bioethics

## Abstract

Charlie Gard, Alfie Evans, and Isaiah Haastrup were infants at the centre of highly publicised court proceedings in the United Kingdom concerning the permissibility of continuing life-sustaining treatment in the face of devastating neurological injury. In each case, the court authorised withdrawal of treatment in favour of palliative care, concluding that the infants should be allowed to die with dignity. The properties commonly thought to ground dignity—such as sentience, rational capacity, or human flourishing—however, were either absent or not threatened by continued treatment, creating an apparent tension in the court’s reasoning: although withdrawal is justified as serving the infants’ dignity, dignity itself appears unable to perform the adjudicative role it was assigned. In this paper, I articulate a novel account of dignity that understands treating a person with dignity as a process of moral reflection and action grounded in recognition respect. I argue that this account can adjudicate the cases of Charlie, Alfie, and Isaiah while preserving the moral assumptions reflected in the High Court rulings: that the infants were worthy of moral consideration, and that continued life-sustaining treatment or experimental intervention would have constituted an affront to their dignity.

## Introduction

Charlie Gard, Alfie Evans, and Isaiah Haastrup were infants at the centre of highly publicised court proceedings in the United Kingdom, each concerning the permissibility of continuing life-sustaining treatment in the face of devastating neurological injury. All three infants had suffered profound and irreversible brain damage, resulting in total or near-total paralysis. Medical experts testified that they lacked the capacity to see, hear, or respond meaningfully to external stimuli and that any experience of pain or consciousness was unlikely. Due to the perceived futility of further intervention and the considerable burdens associated with ongoing invasive treatment, the courts held that continued treatment and life-sustaining measures were not in the children’s best interests and would, moreover, be an affront to their dignity. Each court therefore authorised the withdrawal of life-sustaining treatment in favour of palliative care, concluding that Charlie Gard, Alfie Evans, and Isaiah Haastrup should be allowed to die with dignity [1–3].

Appeals to dignity, and the role accorded to dignity within best-interests assessments in cases such as these, can be problematic. This is because the properties that are commonly thought to ground dignity claims—such as sentience, the capacity for reason, or human flourishing—are either absent or not threatened by continued treatment [4]. The result, in the cases of Charlie, Alfie, and Isaiah, is tension in the courts’ reasoning: although withdrawal of treatment is justified as serving the infants’ dignity, dignity itself does not appear capable of doing the adjudicative work it is asked to perform.

These cases, and the tension that arises, highlight the more general issue that unless an action compromises the grounds of a person’s dignity—or, in some cases, violates the basic rights they hold in virtue of that dignity—objections to that action cannot coherently be framed in the language of dignity at all. This leads to deeply counter-intuitive implications in some cases, such as the abuse of cognitively disabled but willing participants, or the poor treatment of persons in permanent vegetative states. Such actions may all be perfectly compatible with the moral demands that respect for their dignity, or lack thereof, calls for. Of course, different conceptions will yield different normative conclusions, but crucially, on some prominent accounts these actions fail to register as dignity-violations not because they are morally innocuous, but because the theory lacks the conceptual resources to explain why they are wrong in dignity-based terms when grounds remain unthreatened.^1^

In this paper, I argue that one way to adequately explain and adjudicate these hard cases is to reinterpret the moral imperative to treat someone with ‘dignity’ as an appeal to recognition respect; that is, a call to give appropriate consideration to the morally salient features of an object, including persons, in one’s deliberations, and to act appropriately in light of those deliberations [5]. This conception, I argue, *can* adjudicate the hard cases of Charlie, Alfie, and Isaiah, and can do so in a way that preserves the assumptions reflected in the High Court rulings: that the infants were worthy of moral consideration, and that the continuation of life-sustaining treatment or the provision of experimental interventions would have constituted a failure to treat them with dignity. Moreover, because recognition respect already underpins the forms of dignity most commonly ascribed to persons, it also provides a coherent and readily applicable framework for assessing the other hard cases discussed above, extending a mode of moral reasoning already familiar from existing appeals to dignity.

In this paper, I focus primarily on the cases of Charlie, Alfie, and Isaiah. I begin briefly by showing why leading accounts of dignity are unable to adequately resolve the tensions that arise in these cases. I then advance an alternative conception of dignity as recognition respect and show how it can frame the withdrawal of treatment as a requirement of respecting their dignity. I conclude by considering an objection to widening the domain of ‘dignity’ in the ways recognition respect permits, and I defend an inclusive framework capable of addressing the other challenging cases discussed above.

## Hard cases and the dignity dilemma

‘Dignity’ is a complex term with meaning and function operating differently within different contexts. Within philosophical and legal traditions, one common way of understanding ‘dignity’ is as referring to the moral standing of human beings. Broadly, to say that human beings have dignity is to claim that they possess inherent moral value in virtue of some feature, or set of features, they hold. Such value imposes corresponding demands and constraints on how individuals must be regarded and treated. While there is a substantial literature in bioethics distinguishing between ‘moral standing’ and ‘moral status’, for the purposes of this paper I use the terms interchangeably. In the cases of Charlie, Alfie, and Isaiah, the courts invoked the infants’ dignity to argue that continued treatment would violate the moral obligations owed to them in virtue of their moral standing.

The features that ground human dignity are contested and vary across accounts. Common grounds include one or a combination of the following: membership in the species *homo sapiens* [6], sentience [7], the capacity for reason [8], the capacity for mutual recognition and reciprocity [9], relational bonds of love and esteem [10], particularity [11], and capacity for human flourishing [12]. As Jonas and Evans aptly observe, “these conceptions yield different conclusions about Charlie, Alfie, and Isaiah,” yet none clearly supports the courts’ rulings [4]. 

On the one hand, the infants lacked capacities for reason, mutual recognition, and reciprocity, were likely not sentient, and were not flourishing on any objective standard. As such, these features could not plausibly ground appeals to dignity, and thus failed to provide the normative adjudicative force invoked by the courts. On the other hand, the infants were *Homo sapiens*, objects of relational bonds of love and esteem, and were particular human beings. If reciprocity is not required to ground dignity within interpersonal relationships, and if intentionality is not required to ground particularity—that is, an awareness of oneself as a particular individual, or, as Robin Dillon puts it, as a “‘me’ in all my specificity”—then at least three bases for appeals to dignity remain [11]. 2 Even here though, the courts’ contentions are left unsupported.

Depending on how ‘dignity’ is specified, threats to it may arise either from the way one comports oneself (often described as exhibiting a “lack of integrity” or a “lack of self-respect”) or from the way one is treated by others or by broader processes, such as harms arising from systemic injustice. Threats of the former kind often arise when an individual fails to treat themselves in ways that reflect proper appreciation of their own moral status or basic moral rights and duties they possess in virtue of that status. Threats of the latter occur when one is not treated with appropriate regard by others or is harmed by unjust or otherwise problematic social structures. Given their limited capacities, the only plausible threats to dignity in the cases of Charlie, Alfie, and Isaiah were external rather than self-directed.

Typically, when claims are made that a person’s dignity has been threatened externally, the identified failure lies in failing to respect the moral rights a person possesses in virtue of that status, and which serve to protect it. On some interpretations of the Kantian view, to lie to a person, for example, is to fail to treat them with dignity because it directly undermines the very grounds of that dignity—their capacity for agency—and, in doing so, violates the basic moral rights they possess that serve to protect it [13]. 

Similarly, privileging one’s own interests over another’s simply because they are one’s own would also constitute a failure to treat that person (and oneself) with dignity. This is not because it necessarily undermines the grounds of that person’s dignity—though it could—but because it fails to show proper regard for the basic moral rights that persons share equally in virtue of their moral status, including the right to have one’s interests taken seriously and given equal moral consideration. In both cases, the failure consists in acting in ways contrary both to the promotion and protection of agency and to the respect owed to persons as moral equals.

On this understanding, for continued treatment to threaten the infants’ dignity, it would have to undermine the basic moral rights they possess in virtue of their moral status and that serve to protect it—namely, those grounded in their species membership, their relational bonds of love and esteem, or their particularity. It is far from clear, however, how continued treatment might do so, or that those rights would be better served by withdrawal [4]. Indeed, in some respects, the infants’ dignity and basic rights might instead be understood as being *upheld* by continued treatment, insofar as it sustains the conditions under which that status is recognised.

## Agency and undignified lives

Given that these common grounds for dignity do not support the judicial rulings, why do the courts nonetheless appeal to the concept? One explanation offered by Jonas and Evans is that the courts are working with a different conception of ‘dignity’: one that imposes a form of ‘capacity dignity’ into the evaluation of human life [4]. On their reading, the capacity for agency is treated as foundational to human dignity, such that a life without this capacity is regarded as undignified, not worth living, and so not in the infant’s best interests to preserve. An alternative view that Jonas and Evans offer is that “because they had no discernible capacities, these infants had no discernible interests upon which to base a best-interests’ assessment on.” [4].

To address the complexities of these hard cases, and others like them, it is my view that one need not—and indeed *should* not—conclude that the infants have no interests. At the very least, the fact that a capacity to experience pain cannot be ruled out is undoubtedly a morally relevant consideration. There are also views within the philosophical tradition that hold that all living entities have interests, even in the absence of capacities for experience or cognition, although I will not explore them here.^3^ I do agree, however, with Jonas and Evans’s general point that it makes little sense in this context to attribute to these infants an interest in a capacity they can never realise, the absence of which causes them no suffering. Nor is it clear that withdrawal of treatment better serves any such supposed unrealisable interest. If this is indeed the position of the courts, it is clear that a shift in thinking must take place.

## Rejecting dignity?

Many theorists have rejected the utility of appeals to dignity in ethical assessment, and not just in hard cases such as these. Ruth Macklin notably claims that “dignity is a useless concept,” and that it “means no more than respect for persons or their autonomy.” [16] Doris Schroeder and Suzy Killmister offer responses to Macklin arguing for the utility of the concept, disentangling it from the ambiguities that Macklin contends are partly to blame [17, 18]. In line with the Kantian tradition, Killmister, for example, argues that “dignity is the inherent capacity for upholding one’s principles.” [18] The source of one’s dignity here is this capacity, and its exercise—as well as that which can be undermined—is the expression of that dignity. On Killmister’s view, “individuals for whom such reasoning is impossible”—including infants such as Charlie, Alfie, and Isaiah—are not excluded from moral consideration [18]. They are, however, not the kind of beings to whom ‘dignity’ is properly ascribed, since dignity refers only to those capable of the relevant form of autonomous, self-reflective reasoning.

While Schroeder and Killmister articulate important uses of ‘dignity’ when referring to agents, it is my view that ‘dignity’ can also extend to non-agents in ways that are helpful for adjudicating the hard cases, and other hard cases involving persons who do not properly count as agents.^4^ Moreover, it can do so without collapsing dignity into the reductive terms Macklin criticises, and without relocating moral significance of dignity exclusively in the dignity of external parties—as a flourishing-based account such as Charles Foster’s would require [12]. One way to do so, I propose, is to understand the demand to treat someone with ‘dignity’ as an appeal to recognition respect.

## Dignity as recognition respect

### Darwall on recognition respect

Dignity and respect are closely related concepts, so much so that the terms are often used interchangeably in ordinary discourse. Within the philosophical tradition, however, an important distinction is often made: dignity is typically understood as a property an entity—paradigmatically, a person—has, whereas respect is something a person does or is disposed to do. As such, dignity can ground moral status—or, more precisely, an object is often said to have moral status, characterized as ‘dignity,’ in virtue of some conferring or grounding property—and respect is the appropriate response to that status or property.

Stephen Darwall refers to this form of respect response as “recognition respect.” [5] On his account, recognition respect is “a disposition to weigh appropriately some feature or fact in one’s deliberations.” [5] Objects of recognition respect include persons as such, persons in particular roles, feelings, institutions, nature, and so on. To respect something in this way is just to “regard it as something to be reckoned with (in the appropriate way) and to act accordingly.” [5].

Moral recognition respect is a narrower form of recognition respect. When one has moral recognition respect, the disposition one assumes is a *moral* attitude rather than, for example, a purely prudential one. To respect an object in this way is to “regard it as requiring restrictions on the moral acceptability of actions connected with it.” [5] Some fact or feature is an appropriate object of moral recognition respect, on this view, “if inappropriate consideration of weighing of that fact or feature would result in behaviour that is morally wrong.” [5] To say that persons as such are due moral recognition respect is to say that “they are entitled to have other persons take seriously and weigh appropriately the fact that they are persons in deliberating about what to do.” [5].

What moral recognition respect demands varies depending on one’s moral commitments and theories. According to Darwall’s interpretation of the Kantian tradition, for example, recognition respect for persons—and so treating someone with dignity, as they are, here, one and the same—calls for persons to respect the capacity that grounds dignity. The dignity of persons, on this view, “consists, not just in requirements that are rooted in our common nature as free and rational, but also in our equal authority to require or demand of one another that we comply with these requirements”—that is to “occupy the second person standpoint.” [20] In broad terms recognition of these features requires non-interference (a perfect moral duty) with and support (an imperfect moral duty) for a person’s agentic capacities, as well as respect for the basic moral rights they hold in virtue of that status. Basic rights typically include a right to fair and equal treatment among persons, the right to have one’s preferences and interests taken seriously and weighed appropriately in the deliberations of others, and the right to be understood as having the authority to make claims and issue demands for these basic rights [5, 9]. 

Importantly, even when directed toward an individual’s basic moral rights, recognition respect remains a relational form of valuing. It requires acknowledging that persons are situated within interpersonal and other kinds of relationships, and that giving these facts appropriate moral weight involves situating them within—and weighing them against—other relevant considerations. Here then, respect for a person’s dignity is not an independent consideration to be balanced against others, but the act itself of deliberating and acting appropriately in light of the morally salient facts.

This feature—dignity as consideration and action—while underpinning many accounts of dignity, also distinguishes it from views that treat dignity either as one factor among others to be weighed in, for example, a utilitarian calculus [12], or as a constraint on action, where dignity is understood as incommensurable and inviolable [21]. 

### Extending recognition respect beyond agents

Darwall’s Kantian picture of dignity as recognition respect situates agency and the ability to occupy the second person standpoint as foundational to moral recognition respect. As such, this form of recognition respect is unhelpful for adjudicating the cases of Charlie, Alfie, and Isaiah. However, recognition respect need not be so narrowly defined. Darwall himself claims that other entities, including the law, can be objects of moral recognition respect: “to have such respect for the law, say, is to be disposed to regard the fact that something is the law as restricting the class of actions that would be morally permissible.” [5].

There is also a distinction between (i) what makes a fact or feature morally relevant, and (ii) what it is to respond to such facts with recognition respect. On Darwall’s account, recognition respect does not determine which facts are morally relevant; rather, it specifies that there are appropriate ways of responding to entities and facts that have moral value or are morally salient. What counts as an entity that is worthy of moral consideration, or a morally salient fact is determined by one’s broader moral commitments or theory, rather than by recognition respect itself.

This may seem like a weakness of the theory: how can recognition respect adjudicate any case, let alone hard cases, if it does not specify what counts as an appropriate object or which facts are relevant to its consideration? It is my view, however, that the flexibility of the framework is a strength. To answer the question of what has moral status, and which features should count as morally salient, one need not posit any metaphysically suspicious facts—such as identifying some feature or set of features that ground an entity’s supposed special moral status, like “personhood” or “species membership.” Instead, one can look to practice and build from there.

Within bioethics, the law, and many other domains, for example, it is widely accepted that patients, regardless of their ability to occupy the second-person standpoint, are owed moral consideration. It is also widely accepted that there are facts about patients that it would be morally impermissible to disregard. These include patients’ vulnerability to harm, their potential for benefit, and considerations of justice, which require attention to their particular needs and to the fair distribution of resources [22]. Moreover, in decision-making for minors, parental interests and preferences are also taken to be morally salient [23], as are interpersonal bonds of love and esteem between patients and their loved ones [10]. 

Insofar as one endorses this position, recognition respect requires that these facts be appropriately considered and acted upon in the cases to which they apply. On this account, patients (like persons, on Darwall’s Kantian view) are the primary objects of recognition respect, and what that respect requires is determined by the morally salient facts about them. A failure to consider and act on such facts appropriately then constitutes a failure to treat the patient with recognition respect.

### Why ‘dignity’?

If we grant that objects other than persons can be objects of recognition respect, one might still ask why this form of respect should be characterised specifically in terms of ‘dignity’. As I discuss in more detail later, ‘dignity’ has traditionally been reserved for agents and is taken to mark something uniquely valuable about persons as such.

According to Kant, the special status persons have, and the view that they are the proper objects of dignity, is made evident to us in the reverence we have towards persons as such.^5^ He says:

“This idea of personality [that is, freedom and independence from inclination and the capacity to be governed by reason], awakening respect by setting before our eyes the sublimity of our nature (in its vocation) … is easily observed.” [24].

The respect that is awakened in us when observing our nature is multifaceted. It first involves respect as a reverence of sorts for our nature (*Achtung reverentia*) that is a “feeling of a special kind” which is at the same time, but not equivalent to, a sense of awe and fear; awe at our capacity for freedom and independence (our legislative sovereignty), and fear of our finitude with respect to our capacity to curb inclinations in line with the proper respect called for by our nature [25]. The second is the practical response to that awe, which is to engage in recognition respect.

I take dignity to be the appropriate description of the kind of recognition respect owed patients, including Charlie, Alfie, and Isaiah, because *reverence* is the moral attitude befitting them, as it is all persons and all life. Importantly, this does not mean that all life must necessarily be preserved, but it does mean that life must be treated with the reverence and respect in our deliberations and actions that is befitting it.

### Clarifying theoretical aims

Before proceeding to an analysis of Charlie, Alfie, and Isaiah’s cases, it is important to clarify the theoretical aims of the account I am putting forward here.

The account that I offer here is not primarily an interpretation of Kant himself, nor is it an internal revision of a Kantian account of dignity. It is also not an attempt to reconceptualise agency itself. Rather, I draw on Darwall’s notion of recognition respect to articulate a wider framework for understanding dignity—one that is not restricted to agency as its sole ground. In this respect, the view advanced here is best understood as a form of post-Kantian conceptual rearticulation: it retains Darwall’s account of recognition respect as specifying that there are appropriate ways of responding to independently morally salient facts, as well as the Kantian notion of dignity as tied to proper objects of reverence, while extending its application beyond agents. In doing so, this framework is used to reinterpret dignity claims in contexts where agency is absent or unable to do the required normative work.

In terms of adjudicative power, the account I offer does not provide a decision procedure or fixed metric for resolving competing claims. It does, however, supply a standard by which deliberation and its outcomes can be assessed: it requires that the morally salient features of a case be appropriately recognised, weighed, and acted upon. Where those features are sufficiently clear and widely accepted, the framework does not merely structure deliberation but supports clear adjudication. The account does not, however, eliminate disagreement in more contested cases, where there is dispute about which facts are morally salient or how they should be weighed. This is not a limitation unique to the present view, but a feature of moral reasoning more generally.

Before turning to consider the cases at hand, it is important to note that Kantian views differ on the moral status of infants, potential agency, and severely cognitively impaired human beings. Some theorists, such as Patrick Kain, argue that because Kant claims that “all human beings have dignity or are ends in themselves,” all human beings, including those with severe cognitive disabilities, “necessarily have the requisite features of moral personhood.” [26, 27] If moral personhood demands respect for dignity, then, on this interpretation, infants such as Charlie, Alfie, and Isaiah should be treated with dignity. Importantly for present purposes, however, this kind of account does not explain why ceasing treatment may itself count as a way of treating such infants with dignity.

## Recognition respect in the hard cases

### Recognition respect in the hard cases: first pass

Unlike the common accounts of dignity articulated earlier, a framework of dignity built upon recognition respect can accommodate a call to respect the dignity of Charlie, Alfie, and Isaiah.

The infants, as I have said, are not the appropriate objects of recognition respect as persons as such, if this is understood in agency-based terms. Nonetheless, insofar as one agrees that the infants, and so the facts about them, are due moral consideration, and that the appropriate moral attitude towards them is one of reverence—a position that is widely held and fundamental to the courts’ reasoning—it is evident that Charlie, Alfie, and Isaiah are due dignified treatment in the form of recognition respect.

Given the relevant facts, as I articulate in Table 1, it is also evident that continued treatment would constitute a failure to treat the infants with dignity.

**Table 1:** Facts relevant to recognition respect in hard cases

1.	The infants sustained neurological injuries that rendered them incapable of experiencing meaningful interaction, developmental progress, sensory capacities (esp. hearing and vision).
2.	The injuries were judged, based on a strong and explicit multi-expert consensus, to be irreversible and treatment futile.
3.	Clinicians could not rule out that the infants experienced pain associated with ongoing or invasive treatment.
4.	Charlie and Alfie experienced seizures, and Isaiah had epileptic encephalopathy with ongoing seizure risk.
5.	All three infants lacked airway-protective reflexes and required suctioning and invasive ventilation.
6.	The infants could not survive without continued life-sustaining care.
7.	The infants were in relationships with persons who loved and cared for them deeply, despite their inability to reciprocate.
8.	The infants stood in relation to persons who held a professional duty of care to avoid causing harm.
9.	Continued treatment and intensive support would place burdens on the healthcare system.

These facts are relevant as they bear on patients’ vulnerability to harm, their potential for benefit, and considerations of justice, as well as parental interests and preferences and the bonds of love and esteem between patients and their loved ones. As argued earlier, such considerations are widely recognised as morally impermissible to disregard. Recognition respect therefore requires that they be appropriately considered and acted upon in deliberation.

Among these facts, one of the most salient in assessing respectful treatment in these cases is the fact of futility. If, as the expert medical consensus established, the infants’ conditions were such that further treatment could not benefit them, then, other things being equal, providing treatment would constitute a failure of practical reasoning: a failure to reason appropriately in light of the relevant facts. As described above, recognition respect requires giving facts their proper deliberative weight and acting accordingly. Treating the infants despite the futility of treatment would therefore amount to a failure of recognition respect in this practical sense: it would involve pursuing a benefit for the child by means that are believed to be incapable of realising it.

However, the significance of this failure is not merely practical. Where treatment imposes burdens (such as pain, invasive intervention, and physiological stress) without any corresponding prospect of benefit, it constitutes harm without sufficient justification. Under widely accepted and fundamental bioethical principles, it is morally impermissible as it fails to uphold the duty of non-maleficence.

I argued earlier that, on the most common conceptions of dignity, for continued treatment to threaten the infants’ dignity it would have to either compromise the very grounds on which that dignity rests or violate the basic moral rights they possess in virtue of that status—rights that serve to protect and promote that status. Failing to uphold the duty of non-maleficence does not obviously do either in these cases. This is because, while such actions are morally problematic, they do not undermine the grounds that confer dignity on these views.

On the view that treating an individual with dignity consists in moral recognition respect, however, a failure to uphold the duty of non-maleficence is appropriately characterised as a violation of the infants’ dignity—at least on a first pass. This is not because such treatment attacks the grounds of dignity, but because ongoing treatment fails to give appropriate weight to the morally salient features of the infant and their situation, including their vulnerability to harm, the absence of any prospect of benefit, and the burdens imposed by continued intervention.

### Recognition respect in the hard cases: distinguishing recognition respect

While the initial analysis tells against continued treatment, a proper adjudication requires attending to further considerations. Before turning to these, however, several clarificatory distinctions are in order.

Principles such as non-maleficence and best interests, as well as professional obligations and respect for parental concern, are familiar in bioethical reasoning. Many of the considerations identified in Table 1 also overlap with those invoked in quality-of-life (QOL) assessments, such as the infants’ capacity for pain or their lack of developmental prospects. Considerations such as dependency, vulnerability, and interpersonal relationships likewise figure in well-established feminist theories, including relational accounts of dignity [28], care ethics [29], and in vulnerability-based approaches to ethics [30]. As such, one might rightly wonder, at this stage, what distinctive normative contribution dignity as recognition respect has to offer.

Recognition respect, however, though it engages with overlapping considerations, is not reducible to any of these approaches or values, nor to what we might call “ordinary moral deliberation.”

Consider, first, QOL and best-interests approaches to bioethics. These approaches typically assess the permissibility of treatment by evaluating the patient’s expected experiential welfare, often focusing on whether continued life would be beneficial, harmful, or worth living. By contrast, the account developed here understands such considerations as part of a broader set of morally salient facts that must be appropriately recognised, weighed, and acted upon. These include not only welfare-related features, but also relational and role-based facts—for example, that the infants stand in relationships with others who love and care for them, despite their inability to reciprocate, and that they are the objects of professional duties of care. Recognition respect, then, is not a metric for assessing the value of a life or its interests, but a framework for responding appropriately to the features of an individual in the given circumstances.

The same is true of relational accounts of dignity, care ethics, and vulnerability-based approaches to ethics. While these rightly emphasise the moral significance of dependency, vulnerability, and interpersonal relationships, they differ in both structure and ambition. Recognition respect does not treat care or relationality as foundational or dignity-conferring values. Rather, such considerations are understood as among the features that must be appropriately recognised and weighed in deliberation. As such, relational-, care- and vulnerability-based considerations, like those associated with QOL and professional obligations, are not displaced but incorporated: they help to identify what matters morally, while recognition respect specifies what it is to respond to those considerations appropriately.

The distinctive feature of dignity as recognition respect, however, is not that it asks what one ought to do, all things considered, as a general moral theory might, or what the “wrong-making” feature of a given action is. Rather, it frames deliberation and action in terms of what it means to show a particular individual proper respect—that is, to treat them with dignity, regardless of their capacity for agency.

While familiar values and approaches bear on this question, none takes dignity as its central organising concern—or, if they do, they are not of the kind that are threatened by the facts of the present cases. On the account presented here, the moral task is then, is not simply to gather familiar moral considerations under one unifying frame or to engage in ordinary moral deliberation. It is to deliberate, evaluate and act appropriately with those considerations in mind, and to do so with the individual firmly in view. It also requires that this deliberation be guided by an appropriate moral attitude, namely one of reverence for the individual. Even where ordinary moral deliberation might arrive at the same outcome, it lacks the distinctive focus, orientation, and intentionality that treating someone with dignity as recognition respect provides.

The value of this approach, in terms of the normative work it performs, is that it preserves the moral question of what respectful treatment demands even when other accounts—such as agency-based or interest-based accounts—fail or become difficult to apply. Because dignity as recognition respect is responsive to considerations of agency, welfare, and determinate interests, without being grounded exclusively in any one of them, the question of respect remains clearly in view even when a particular feature is absent, diminished, or difficult to determine. This allows us to assess not merely whether a given form of treatment is permissible or impermissible, but whether it is respectful: whether it keeps the patient at the centre of deliberation and responds to them in a way that reflects the moral regard they are due.

With this in mind, let us now return to the cases at hand.

### Recognition respect in the hard cases: qualifications and considerations

While I argued that initial consideration tells against continued treatment of Charlie, Alfie, and Isaiah, there remain important considerations and qualifications:

First, in each of the hard cases, the infants were loved and cared for deeply by their parents. Their parents also advocated strongly for the continuation of their children’s care and treatment, as well as commitment to sustained support of that care. Isaiah’s father, for example, states that “I can come here for the next five years. Coming here every day is not a burden.” [3] All parents also believed that further treatment would not be futile—Isaiah’s mother, for example, says “I do respect the doctors’ opinion, I am not saying they are completely wrong, but I want them to take into account my views and what I have seen.” [3].

While recognition respect calls for us to consider specific facts about the infants, it is also inherently relational; it demands that we acknowledge that the infants, like all persons, are situated within relationships, and that giving appropriate consideration to the facts about the infants requires situating those facts within, and weighing them against, other pertinent and morally relevant facts, values, interests, and claims. Given that parental autonomy and bonds of love and care are widely recognised as values that it would be impermissible to disregard, these facts too must weigh into the determination of the demands of recognition respect—that is, as a matter of respect for the children, and for the parents too.^6^

Second, expert testimony emphasised that the infants’ ability to experience pain could not be ruled out, while also stating that it was unlikely that the infants experienced consciousness. Consciousness is necessary to experience pain as suffering, and it is typically suffering, not pain itself, that is considered morally objectionable. Charlie, Alfie, and Isaiah exhibited physical responses to potentially uncomfortable or painful stimuli, such as limb withdrawal, stiffening on touch, or reactions to injections [1–3]. However, clinicians stated that these responses were likely reflexive or seizure-related and therefore not indications of conscious pain [1–3]. 

And third, while continued treatment was considered futile in all three cases, Charlie Gard’s parents were seeking nucleoside therapy, an experimental and unproven treatment for Charlie’s condition, mitochondrial DNA depletion syndrome (MDDS) [1]. The neurologist, Dr Michio Hirano, offering this experimental treatment to Charlie stated that “I can understand the opinions that he is so severely affected by encephalopathy that any attempt at therapy would be futile. I agree that it is very unlikely that he will improve with that therapy.” [1] As Julian Savulescu emphasises, “very unlikely” means that there is a “very small but non-zero chance of some improvement.” [31].

### Recognition respect in the hard cases: reconsidered

On any plausible moral theory, the risk of causing a child pain, even if very unlikely, with *no* prospect of improving their condition would not be outweighed by parental preferences to proceed. The same is also true for the satisfaction of other interests such as those of the healthcare providers or the potential benefits to science of pursing experimental care. Treating a child in this way would not only be a failure of appropriate regard for their vulnerability, but would also risk endorsing a broader and problematic principle: that it is permissible to use children as mere instruments for personal, institutional, or societal purposes—a principle that runs deeply in opposition to widely endorsed bioethical principles, and threatens the dignity of all persons [22]. 

With respect to testimony, hearing parents’ accounts of their child’s condition is not only appropriate but required as a matter of recognition respect for the parents as agents and caregivers. Parents are often uniquely placed to observe their child over extended periods, particularly in settings such as neonatal intensive care, and may notice behavioural patterns or responses that are not readily apparent to clinical staff. Such observations can provide valuable evidential input. For example, in identifying possible signs of discomfort, responsiveness, or changes over time.

However, the interpretation of these observations, such as what they indicate about pain, consciousness, prognosis, or the likely benefits of treatment, requires clinical expertise. As such, questions concerning diagnosis, prognosis, and the expected effects of intervention rightly fall within the domain of medical judgement. Parental testimony therefore plays an important evidential role, but it does not by itself carry the kind of epistemic authority that can override medical expertise regarding the benefits and burdens of treatment.

As noted earlier, Charlie’s case is unique here because, according to some, there was a “non-zero” chance it might benefit him. Given this chance and the ability of Charlie’s parents to fund his treatment, Savulescu contends that there were no legitimate moral grounds for denying “Charlie Gard his chance of life.” [31] Here then, the argument for dignity might be this:

“Treating Charlie with dignity requires recognising his potential for recovery and according that consideration greater weight than the risk of pain. Under this interpretation, dignity not only permits but positively demands the pursuit of experimental treatment.”

It is important to note, however, that the experimental treatment was not promising Charlie his, albeit very unlikely, “chance of life” or anything like recovery. According to Hirano the experimental treatment offered only a (very low) potential to “improve his condition,” which is not the same as offering a “chance at life” or recovery [1]. Nucleoside therapy had shown some success in preventing seizures and improving health in a variant of MDDS (TK2), but not in Charlie’s variation (RRM2B) and not in repairing seizure-related brain damage—damage that medical experts testified was widespread and irreversible [1]. According to Hirano, a reasonable expected improvement would be improved strength to reduce time on ventilators, “but he agreed that the effect on brain function would be less or minimal or non-existent.” [1] The conclusion drawn was that “While there were some reasons to be hopeful that it might make a modest difference to life expectancy, it almost certainly could not undo structural brain damage.” [1] What the treatment offered then to Charlie—what amounts to his “chance of life” in Savulescu’s opinion—was a very low chance of a slightly longer life with a little less time on ventilators.

Of course, while the chances of brain regeneration were “close to zero as makes no difference,” it is indeed not zero [1]. One might think then that this chance is worth pursuing, especially for one’s child, and perhaps even demanded as a matter of respect. As Dominic Wilkinson explains, however, there are moral limits on what one can permissibly pursue for one’s child, and one of those limits is pain [23]. According to the harm threshold test in paediatric ethics, parental decisions may be overridden where they expose a child to a significant risk of serious harm [32]. 

As described earlier, the court recognised that “no one can be certain whether or not Charlie feels pain.” [1] The court, however, did not specify the potential nature of that pain, either in Charlie’s condition at the time or as a possible consequence of the experimental treatment. If pain were to occur, might it be severe or only mild? Would it likely be continuous or recur across multiple treatment sessions, or would it be short-lived? These questions matter deeply for the permissibility of treatment.

To proceed with treatment without answers, on the basis of only a minimal prospect of benefit, and without being able to rule out severe and prolonged pain, places the child at significant risk of harm that would be both imprudent and morally unjustified.

## The adjudicative power of recognition respect and best-interest assessments

I have argued that the account developed here does not offer a decision procedure or fixed metric for resolving competing claims, but instead provides a standard against which both deliberation and its outcomes can be assessed. Where the morally salient features are sufficiently clear and broadly agreed upon, this standard is capable of yielding clear and determinate judgements. I have also argued that continued treatment would expose the infants to a risk of harm without a corresponding prospect of sufficient benefit and is, on that basis, morally unjustified.

What has not yet been addressed is that discontinuing treatment would, and did, result (in a sense) in the deaths of the infants. This fact is unquestionably morally significant in determining what dignified treatment requires. What, then, does treating someone with dignity demand in these cases?

It would seem that the answer to this question depends on one’s position regarding a set of further and notoriously difficult questions: whether, for example, death can ever serve an individual’s interests such that one might be “better off dead”, or whether life, however minimal its content, ought to be preserved irrespective of cost or burden [33, 34]. 

The account of dignity advanced here—like other accounts of dignity and the principles considered alongside it—does not itself resolve these deeper disputes. It can, however, adjudicate the hard cases once a position on these underlying questions is in place.

For those who, as I do, hold that exposing an infant to the risk of significant pain under such conditions is unjustified and impermissible, continued treatment would constitute a failure to treat the infants with dignity. I hold this view not because it would necessarily fail to be in the child’s best interests, or because death is necessarily better than a life in pain. Rather, it is my view that others—including a child’s parents—are not permitted to choose a life of pain for a child, or to risk such a life in the face of minimal benefit.^7^ To take on such burdens requires consent, and it is a fact of the matter that consent is beyond what the children can provide. Recognition respect therefore requires that such burdens not be imposed.

## The contributions of dignity as recognition respect

At the beginning of this paper, I set out to show how the moral imperative to treat someone with “dignity” might be reinterpreted as an appeal to recognition respect—that is, as a call to give appropriate consideration to the morally salient features of an individual and to act accordingly. I have argued that this conception can adjudicate the hard cases of Charlie, Alfie, and Isaiah in a way that preserves the central assumptions reflected in the High Court rulings: that the infants were worthy of moral consideration, and that continued life-sustaining treatment or experimental intervention would have constituted a failure to treat them with dignity.

The central contribution of this account, I have argued, is that it preserves the moral question of what respectful treatment demands even when other familiar accounts fail or become difficult to apply. It does so by locating the demands of dignified treatment not in the possession of any particular capacity, but in the requirement to respond appropriately to the morally salient features of an individual, regardless of agency. In this respect, it departs from agency-based accounts [18, 35–37] and from other non-agential views [10, 28]. It also remains responsive to familiar considerations of welfare, harm, vulnerability, care, relationality, and professional obligation, without being reducible to them.

On this view, treating an individual with dignity is not primarily a matter of protecting or promoting a status or property. It is a mode of moral attention and action: a way of deliberating and acting that keeps the individual, and the facts about them, firmly in view. It also requires an appropriate mode of regard (*reverence*) through which those facts are recognised and given the weight they are due.

This shift has practical as well as theoretical significance. It explains how appeals to dignity in the cases of Charlie, Alfie, and Isaiah can be both intelligible and justified, without relying on controversial claims about the value of their lives or the erosion of some underlying dignity-conferring property. Continued treatment would have constituted a failure of dignity not because their lives lacked value, but because, in the circumstances, it would have failed to respond appropriately to the morally salient facts about them.

More broadly, the account shows how dignity can function not as a residual or rhetorical concept, but as a substantive guide to moral deliberation in some of the most difficult cases in bioethics. It does not itself resolve all of the deeper disputes on which such deliberation ultimately depends, but it clarifies what is at stake in treating someone with dignity: not merely reaching a permissible outcome, but responding to the individual with the moral regard they are due.

## The demands and status of dignity

As the account of dignity proposed here extends the scope of dignified treatment beyond agency-based accounts, it is open to a familiar objection: the risk of “seeing dignity everywhere.” As others have noted, dignity is often invoked to justify a wide range of, at times conflicting, claims [12], leading some to dismiss it as a useless or vacuous concept [16, 38]. One might worry that the present view, in extending dignity beyond agents, exacerbates this concern [39]. 

The core of the objection is that if dignity applies too broadly, it risks losing its normative distinctiveness. In particular, extending dignified treatment to non-agents may undermine the idea that dignity marks a special moral status—one that grounds heightened or distinctive claims in the case of *persons*. As Remy Debes articulates the point: “a proper account of dignity must pick out a distinctive value belonging to human beings,” though he also notes that “this is not equivalent to demanding a value that belongs distinctively to humans.” [21].

The worry, then, is that by extending dignity in this way, the present account risks diluting both its normative force and its capacity to ground the special claims typically associated with persons.

The attribution of a distinct value of or special moral status held by persons is a recurrent theme in religious, philosophical, literary, and artistic traditions across cultures and historical periods [40]. 8 It is therefore unsurprising that this idea occupies an established place within the dignity literature. Tradition alone, however, does not justify conceptual constraint. Where inherited notions fail to perform the normative work demanded of them—as the hard cases vividly illustrate—there is no reason to preserve them merely for the sake of continuity. Moreover, as I claimed at the beginning, some leading accounts of dignity that aim to pick out grounding features that belong to persons lead to deeply counter-intuitive conclusions, especially for those who are vulnerable to harm.

It is my view, however, that we do not require a special category, status, or value for persons in order to capture what is valuable in traditional accounts of dignity. Such accounts rightly maintain that persons are worthy of moral consideration and that there are morally salient facts about them—their agency, particularity, or bonds of love and esteem, etc.—that demand appropriate consideration in deliberation and action. What the view I have presented here amounts to—borrowing a colloquial expression—is an inclusive “yes, and” position: yes, persons are worthy of moral consideration, and there are facts about them that call for appropriate regard; but these facts should not be limited to the properties thought to ground a person’s moral status, nor necessarily to the properties of persons at all.^9^

A virtue of this expansive view, I have argued, is that it helps to clarify why the hard cases of Charlie Gard, Alfie Evans, and Isaiah Haastrup raise genuine and pressing questions about how to treat the infants with dignity.

A further virtue of this view is that it clarifies what is morally troubling as a matter of dignity about other hard cases, such as those introduced at the outset, and does so without requiring recourse to the dignity of third parties. The abuse of cognitively disabled but willing participants, or the mistreatment of persons in permanent vegetative states, affronts the dignity of those affected not—or not only—because such actions conflict with the grounds of their dignity or violate their basic rights, but because they manifest a failure to give appropriate regard to the individual’s physical and cognitive vulnerability.

An individual’s vulnerability marks conditions in which they are especially exposed to harm, less able to protect or advance their own interests, and more susceptible to forms of use that track asymmetries of power and dependence. To disregard such features is to license forms of treatment that predictably result in harm, facilitate exploitation, or instrumentalise the individual’s body or condition. These are paradigmatic ways of wronging another. Recognition respect captures this by requiring that such features be given due weight in deliberation; where they are not, the resulting failure is not merely a mistake in judgement, but a moral failure in how the individual is regarded and treated.

A person who abuses a cognitively disabled individual fails to regard that individual’s vulnerability appropriately as a moral constraint on their actions and instead, in many cases, treats it as a fact to be exploited. In doing so, the abuser violates the demands of recognition respect, and thereby the dignity of the person subject to the abuse.

On the account I have articulated here, then, there is no need to posit some special feature that persons possess which itself demands recognition, nor to defend a catalogue of basic rights derived from that status. An appeal to the more basic moral idea that vulnerability warrants due consideration is, and should be, sufficient.

Some may remain dissatisfied, insisting that recognition respect cannot be another name for ‘dignity’ because it only properly applies to persons. The burden of proof, however, lies with such critics to explain not only why the scope of dignity should be confined in this way on grounds other than mere tradition, but also how dignity can be compromised in the hard cases when the moral grounds of that dignity remain untouched.

It is my view that the term ‘dignity’ carries the appropriate degree of reverence for the children and others described in these hard cases, and that dignity, understood as recognition respect, provides the right normative guide.

## Data Availability

No datasets were generated or analysed during the current study.

## Footnotes

1 To explain cases like these, accounts often appeal to the dignity of others as the locus of harm; spitting on someone with a severe cognitive disability, for example, is cast as wrong on agency- or flourishing-based accounts because it constitutes an affront to the dignity of the abuser, and because it may encourage similar assaults on the dignity of others cognitively abled persons.

2 In cases such as these, one might plausibly maintain, following Foster, that continued treatment compromises the dignity of others in a range of respects. Judicial reasoning, however, is directed squarely at the dignity of the infants.

3 Paul W. Taylor [14] , for example, argues that all living things have moral standing and interests worthy of moral consideration. On Philippa Foot’s [15] view too, in so far as things can be good or bad for an organism (such as plants, insects etc.) in a teleological sense, then one might say that organism has interests towards their flourishing.

4 Philosophical accounts of personhood often distinguish “persons” from “human beings,” defining the former in terms of agentic capacities that not all human beings possess [19]. By contrast, ordinary usage treats “person” and “human being” as interchangeable. I adopt the latter usage here, employing “person” to refer to human beings regardless of their agency.

5 Note, too, that Kant himself appeals to a kind of practice—namely, our reverence for persons—as evidence that persons ought to be treated with respect: we revere persons and so should treat them with reverence.

6 Other relevant relational facts might also include the healthcare providers interests, potential burdens on the healthcare system, and, more controversially, the potential benefits to science of pursuing experimental care.

7 It is true that parents are often permitted to pursue treatment they deem to be in their child’s best interests, even without consent; however, such cases rarely involve the kind of imbalance of benefits and burdens present here.

8 For ease of expression, I refer here simply to ‘persons.’ More precisely, some views hold that persons, understood as agents, possess a special moral status or feature that warrants recognition. Other views instead take membership in the species *Homo sapiens*—without requiring the capacity for agency—to confer a special moral status or feature.

9 Such as facts, for example, about distributive justice, or the interests of non-human animals.
